# Sensorimotor integration and motor learning during a novel force-matching task in young adults with attention-deficit/hyperactivity disorder

**DOI:** 10.3389/fnhum.2022.1078925

**Published:** 2023-01-05

**Authors:** Heather S. McCracken, Bernadette A. Murphy, Ushani Ambalavanar, Cheryl M. Glazebrook, Paul C. Yielder

**Affiliations:** ^1^Faculty of Health Sciences, University of Ontario Institute of Technology, Oshawa, ON, Canada; ^2^Faculty of Kinesiology and Recreation Management, University of Manitoba, Winnipeg, MB, Canada; ^3^Health, Leisure & Human Performance Research Institute, University of Manitoba, Winnipeg, MB, Canada; ^4^Faculty of Health, School of Medicine, Deakin University, Waurn Ponds, VIC, Australia

**Keywords:** somatosensory evoked potentials (SEPs), attention-deficit/hyperactivity disorder (ADHD), motor learning, electroencephalography, sensorimotor integration (SMI), force modulation

## Abstract

**Introduction:**

Attention-Deficit/Hyperactivity Disorder (ADHD) is a neurodevelopmental disorder that exhibits unique neurological and behavioral characteristics. Those with ADHD often have noted impairments in motor performance and coordination, including during tasks that require force modulation. The present study provides insight into the role of altered neural processing and SMI in response to a motor learning paradigm requiring force modulation and proprioception, that previous literature has suggested to be altered in those with ADHD, which can also inform our understanding of the neurophysiology underlying sensorimotor integration (SMI) in the general population.

**Methods:**

Adults with ADHD (*n* = 15) and neurotypical controls (*n* = 15) performed a novel force-matching task, where participants used their right-thumb to match a trace template that varied from 2–12% of their Abductor Pollicis Brevis maximum voluntary contraction. This motor task was completed in pre, acquisition, and post blocks. Participants also completed a retention test 24 h later. Median nerve somatosensory-evoked potentials (SEPs) were collected pre and post motor acquisition. SEPs were stimulated at two frequencies, 2.47 Hz and 4.98 Hz, and 1,000 sweeps were recorded using 64-electrode electroencephalography (EEG) at 2,048 Hz. SEP amplitude changes were normalized to each participant’s baseline values for that peak.

**Results:**

Both groups improved at post measures (ADHD: 0.85 ± 0.09; Controls: 0.85 ± 0.10), with improvements maintained at retention (ADHD: 0.82 ± 0.11; Controls: 0.82 ± 0.11). The ADHD group had a decreased N18 post-acquisition (0.87 ± 0.48), while the control N18 increased (1.91 ± 1.43). The N30 increased in both groups, with a small increase in the ADHD group (1.03 ± 0.21) and a more pronounced increase in controls (1.15 ± 0.27).

**Discussion:**

Unique neural differences between groups were found after the acquisition of a novel force-matching motor paradigm, particularly relating to the N18 peak. The N18 differences suggest that those with ADHD have reduced olivary-cerebellar-M1 inhibition when learning a novel motor task dependent on force-modulation, potentially due to difficulties integrating the afferent feedback necessary to perform the task. The results of this work provide evidence that young adults with ADHD have altered proprioceptive processing when learning a novel motor task when compared to neurotypical controls.

## 1. Introduction

Attention-Deficit/Hyperactivity Disorder (ADHD) is described as a neurodevelopmental disorder. The hallmark characteristics associated with ADHD are behavioral signs, such as hyperactivity, impulsivity, and inattention (Visser et al., 2014). These noted behavioral changes can vary in their manifestation, but together have important implications for day to day life. Approximately 11% of children in the U.S. will receive a diagnosis of ADHD (Visser et al., 2014). However, ADHD commonly persists into adulthood, with approximately 65% of those diagnosed during childhood continuing to meet diagnostic criteria as adults (Faraone et al., 2006). How ADHD manifests in adulthood may vary when compared to childhood. Adults with ADHD are noted as having reduced hyperactive tendencies when compared to children (Gentile et al., 2006), potentially due to developing coping strategies in their day to day life. The differences in how signs and symptoms manifest in adulthood may explain why limited literature focuses on ADHD in this age group. This lack of research has resulted in an important cohort being poorly understood. Further research is needed to develop an improved understanding of ADHD symptomology, including underlying neural characteristics. This work advances our understanding of neural processing in neurotypical controls as well as those with ADHD, particularly relating to adult symptomology, which is yet to be fully understood.

While ADHD is defined by behavioral alterations, there are neurological characteristics that are important to note, and that are relevant to the current research. Those with ADHD tend to have reduced cerebral gray matter diffuse throughout the cortex (Proal et al., 2011). With an emphasis on sensorimotor processing brain regions Duerden et al. (2012) assessed cortical morphology of adolescents and adults with ADHD using high-resolution 3D MRI, and cortical thickness using Montreal Neurological Institute (MNI) analyses. Findings included that the pre-supplementary motor area (SMA) in adolescents and the primary somatosensory cortex (S1) in adults were thicker in those with ADHD when compared to neurotypical controls (Duerden et al., 2012). This may have relevance to the sensorimotor alterations reported in individuals with ADHD, including impaired motor and somatosensory processing (Duerden et al., 2012). Of relevance, are the roles that these neural structures have in sensorimotor tasks, and particularly how they may relate to behavioral characteristics in this population. The pre-SMA is highly active in response to learning, particularly during tasks that require hand movements, and has projections to the dorsolateral prefrontal cortex (Nachev et al., 2008). The S1 plays a fundamental role in the processing and integration of incoming afferent somatosensory input, thus contributing to how sensory and motor signals are integrated for the performance of movement, which has clear implications for motor learning and motor control (Borich et al., 2015). Increased thickness in S1 is also noted in other populations with altered somatosensory processing, including those who experience chronic pain (DaSilva et al., 2007). The increased cortical thickness in S1 has been linked to impaired inhibitory processes in populations experiencing pain. Notably, altered inhibition is characteristic of ADHD. Therefore, it is likely that alterations to S1 in those with ADHD may be associated with alterations to motor performance and accuracy, potentially due to inhibitory alterations in S1. Additionally, ADHD is associated with hypoactivation in areas related to sensorimotor functions (Cortese, 2012). One such neurological characteristic is an overall reduction in cerebellar volume, which in recent years has become a potential hallmark for ADHD (Almeida Montes et al., 2011).

The cerebellum is fundamental to the processes underlying motor learning and SMI, including playing a fundamental role in how individuals utilize feedback to refine and control motor output (Koziol et al., 2014). As noted above, the cerebellum is reported to be reduced in size throughout the lifespan in ADHD when compared to neurotypical controls (Castellanos et al., 2002). These cerebellar alterations, in conjunction with common behavioral characteristics, suggests that adaptations to motor learning and motor performance may play an important role in how those with ADHD function in their daily life, including in occupational and educational settings. Many tasks that individuals perform daily, including tying shoes, typing, driving, etc., are all dependant on our ability to acquire new sensorimotor skills. Our ability to acquire these motor patterns will therefore dictate the level of success experienced with these tasks. The ease with which these skills are acquired can be either heightened or impaired as a result of neural function in specific neural structures and circuits. For instance, difficulties in the learning and automating of fine motor skills are strongly related to altered cerebellar function (Koziol et al., 2013). As a task is learned, the performance of said skill typically becomes more automatic in nature (Koziol et al., 2013). This process is regulated by the cerebellum. It is postulated that the behavioral characteristics noted to be associated with ADHD, including hyperactivity and inattention, are a result of, or related to, alterations within fronto-cerebellar circuity (Durston et al., 2011; Koziol et al., 2013). Additionally, the severity of clinical outcomes in those with ADHD are associated with cerebellar volume, as those with greater clinical outcomes have greater reductions in cerebellar volume (Mackie et al., 2007).

Despite the above advances in the understanding of how the structure of the cerebellum may be altered, it is unclear how these neural characteristics will affect processes related to motor learning and performance in adults with ADHD. Utilizing techniques such as somatosensory evoked potentials (SEPs) can provide insight on the level of neural activity within cortical and subcortical structures in response to a novel motor task. SEPs are a non-invasive neurophysiological technique that allow for the assessment of neural structures via stimulation of a peripheral nerve, and are named based on their polarity and latency (Passmore et al., 2014). For instance, the N30 SEP peak, is a negative deflection that occurs 30 ms after stimulation of the peripheral nerve of interest, which will be the median nerve for the current study. The International Federation of Clinical Neurophysiology (IFCN) compiled information for the strategic and standard application of short-latency SEPs (Nuwer et al., 1994). Therefore, SEPs allow for the interpretation of specific neural generators that have been associated with specific peaks (Passmore et al., 2014). Thus, SEPs data can provide pivotal insight into neurophysiological mechanisms. The interpretation of SEP peaks will enhance the current understanding of the neurophysiological processes related to learning motor tasks in adults with ADHD, particularly those tasks that are dependent on force modulation and proprioception.

Although limited literature has addressed motor performance, at either a behavioral or neurophysiological level in adults with ADHD, those with ADHD generally experience difficulties in tasks that require motor coordination and performance (Karatekin et al., 2003; Fliers et al., 2011; Kaiser et al., 2015). As noted above, one such explanation for this may be that those with ADHD have alterations to their inhibitory processes (Feifel et al., 2004). This inhibitory alteration may be a hallmark deficit associated with ADHD (Lijffijt et al., 2005) that manifests as atypical behaviors, including learning new motor skills. Children with ADHD often exhibit difficulties with motor skills, such as handwriting, resulting in poor legibility and reduced speed (Brossard-Racine et al., 2011). Previous work found that those with ADHD exhibit reduced motor performance at retention, measured 24 h after skill acquisition when compared to neurotypical controls (Adi-Japha et al., 2011). This suggests that ADHD may be associated with an impaired consolidation of motor skills. It should be noted that developmental coordination disorder (DCD) has been described as comorbid in as many as 50% of childhood cases of ADHD (Pitcher et al., 2003). However, the prevalence is lower in school groups, with a 35% chance of comorbidity (Miyahara et al., 2001). Although DCD is a unique neurodevelopmental disorder, there is often co-occurrence with other disorders, for example with ADHD and Autism Spectrum Disorder (ASD) (Blank et al., 2019). Due to this, it may be difficult to discern with certainty whether difficulties with motor skills in those with ADHD are related to impulsivity and hyperactivity, or whether in some instances these deficits may be related to comorbid DCD if present. Additionally, deficits in inhibitory force control in young adults with ADHD has been reported when completing a force modulation task (Neely et al., 2017).

Neely et al. (2017) used a Go/No-Go paradigm in which participants utilized the thumb and index finger to grip a load cell calibrated to less than 15% of the MVC of the pinch grip (Neely et al., 2017). Those with ADHD elicited altered force output from their fingertips (Neely et al., 2017). The degree of altered force output was also a predictor for ADHD-related symptoms, showing alterations to inhibitory control in adults with ADHD were present when performing a task dependant on force modulation (Neely et al., 2017). In addition, ADHD was associated with greater and more varied force on the No-Go trials, a result that suggests hyperactivity in the motor systems in conjunction with alterations to inhibitory control mechanisms (Neely et al., 2017).

With respect to motor control, optimal performance is associated with reduced variability (Selen et al., 2006). Therefore, the increased variability in those with ADHD suggest they may experience difficulties with motor skills requiring force modulation. Additionally, force output was associated with ADHD diagnostic criteria (Neely et al., 2017). Neely et al. (2017) suggest that utilizing a force motor task can provide important information on the inhibitory mechanisms evident in this population. Additionally, using such a paradigm in conjunction with neural markers can provide important information on the neural substrates and processing mechanisms that are related to these changes. However, it remains unclear how alterations to force modulation in ADHD will affect their ability to learn and retain a novel motor task dependent on force, which are common to many day-to-day skills.

Many tasks require our ability to modulate force to elicit effective performance. Force modulation depends on proprioception via sensory feedback from several sensory structures, including muscle spindles, golgi tendon organs, Pacinian corpuscles, and the cutaneous receptors of the digits (Schmidt and Lee, 2005). Examples of such tasks in day to day life include applying pressure to a clutch or a gas pedal in a car and using a joystick controller while operating machines. Previous work utilizing a dynamic task requiring force matching of pinch grip, noted activation within brain regions involved in visual attention and proprioception (Brown et al., 2004). Although ADHD is associated with alterations in proprioception (Goulardins et al., 2013; Jung et al., 2014; Alba et al., 2016; Sanz-Cervera et al., 2017), the extent to which alterations to proprioception and force modulation affect motor learning in ADHD is unclear.

Proprioception is defined as ones’ ability to use their senses to understand where their limbs and body are in space and includes the processing of somatosensory input (Alba et al., 2016). Alterations to proprioception is a sensory characteristic that is inherent to ADHD symptomology (Sanz-Cervera et al., 2017). For instance, balance dysfunction is present in ADHD, potentially associated with alterations to proprioception and vestibular function (Zang et al., 2002). It is postulated that alterations to vestibular function and proprioception in those with ADHD are a result of difficulties processing visual information, as visual input acts as a guide to inform body schema and spatial awareness (Jung et al., 2014; Sanz-Cervera et al., 2017). Young boys with ADHD score lower on balance, spatial organization, and fine and global motricity (Goulardins et al., 2013). It is thought that this may be related to delays in peak brain maturation in those with ADHD (Shaw et al., 2007; Goulardins et al., 2013) that impacts efficient and effective sensorimotor integration.

Neurotypical children reach peak cortical thickness by the age of 7.5 years old, whereas children with ADHD reach this milestone by approximately 10.5 years old (Shaw et al., 2007). These maturational delays in the prefrontal cortex have been associated with the altered inhibitory characteristics in ADHD, whereas alterations to frontal regions, including the premotor cortex, are associated with motor planning and performance (Goulardins et al., 2013). Fundamental sensorimotor skills are impaired in children with ADHD compared to neurotypical controls, where many children fall below the 5th percentile range in the fundamental sensory-motor index for their age group (Iwanaga et al., 2006). Additionally, finger localization and tactile discrimination may be impaired in children with ADHD (Iwanaga et al., 2006). Children with ADHD score lower on tests assessing equilibrium, somatosensory function, vestibular function, and visual ratios, which are related to the alterations in balance noted in this population (Shum and Pang, 2009). Taken together, the literature suggesting alterations to motor performance, proprioception, and cortical characteristics in children with ADHD is robust, with important implications for many daily activities. However, due to insufficient literature that currently exists, it remains unclear as to how these motor and neural characteristics may present in adulthood and their influence on motor learning processes.

The purpose of the current work was to assess whether young adults with ADHD exhibit alterations in neural processes related to learning a novel force-matching task (FMT). Utilizing both behavioral and neural variables allows for a multifaceted approach to form an enhanced understanding of motor learning in those with ADHD. The research question that this work aims to address is, do young adults with ADHD experience alterations to motor acquisition and learning when performing a task dependant on force modulation and proprioception? The primary neurophysiological and behavioral variables assessed were short-latency SEP peaks and performance via percent error at each phase of the motor acquisition paradigm. Specific hypotheses include: (1) those with ADHD will exhibit alterations to SEP peaks when compared to neurotypical controls, likely in peaks related to cortico-cerebellar processing; (2) those with ADHD and neurotypical controls will show performance improvements post-acquisition, and based on previous literature, those with ADHD will likely have reduced improvements at retention measures when compared to controls.

## 2. Materials and methods

### 2.1. Ethical approval

Written informed consent was obtained prior to the start of data collection. This study was approved by the Ontario Tech University Research Ethics Board (REB; # 15307). This study was carried out according to the ethical standards set out by the Declaration of Helsinki statutes governing research on human subjects.

### 2.2. Participants

GPOWER statistical software indicated that for a large effect size (*f* = 0.4), an alpha of *p* = 0.05, and a power (1-β) of 0.95 (β set at 0.05 to minimize the chance of a type II error), a sample size of 12 participants per group is needed for a pre-post experimental design (Faul and Erdfelder, 1992). This study consisted of two groups of participants, one group included adults with ADHD (*n* = 15, 9 females, mean age = 22.00 ± 2.51) and one group of neurotypical controls (*n* = 15, 9 females, mean age = 20.80 ± 1.97). Participants completed several pre-screening questionnaires to ensure they met the inclusion criteria, including being between the ages of 18-35 years old, right-hand dominant, the absence of any other known neurological conditions, or history of injury such as concussions. Handedness was confirmed using the Edinburgh Handedness Inventory (EHI) self-report questionnaire.

Each participant completed the adult ADHD Self-Report Scale (ASRS-v1.1). This was used to assess symptoms associated with ADHD in both groups. The ASRS consists of 18 questions, divided into part A and part B, these questions are in line with the ADHD diagnostic criteria set out in the DSM-IV (Dankner et al., 2017). Each of the 18 questions is rated on a 5-point Likert scale ranging from “never” to “very often.” The ASRS tool is highly sensitive to predicting ADHD symptomatology (van de Glind et al., 2013). Scores for part A and part B were recorded for each participant. Although no particular score is associated with a diagnosis, a higher score indicates a greater prevalence of signs and symptoms associated with ADHD. Those in the ADHD group had an average score of 22.40 ± 4.44 for part A, while controls scored 14.27 ± 4.46. The average score for part B for the ADHD group was 44.07 ± 8.16 and 24.93 ± 6.18 for controls.

### 2.3. Experimental protocol

**FIGURE 1:**
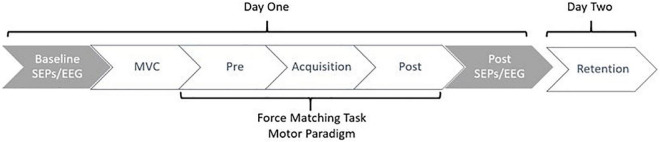
Figure depicting the study flow. Collections occurred over a period of 24-48 h, where participants returned to the lab to complete the retention test 24-48 h after day one. Gray indicates neurophysiological measures, whereas white indicates behavioral measures.

Data collection sessions occurred over two days. All participants attended two sessions, the second being 24-48 h after the first. The first session included EEG and SEP collections, where participants completed the motor task in phases, beginning with the pre-acquisition phase and finishing with the post-acquisition phase of the force-matching task (FMT). On day one, participants completed the informed consent documents, giving both written and verbal informed consent prior to the commencement of the session. This was followed by the setup of the EEG and SEPs. Baseline SEPs measurements were then recorded, including both 2.47 Hz and 4.98 Hz stimulation frequencies. Following baseline SEPs, participants completed the novel force-matching task (FMT) in blocks of pre-acquisition (4-blocks), acquisition (12-blocks), and post-acquisition (4-blocks) as depicted in Figure 1. Each block consisted of 3-5 trials. Post-SEP measurements were then recorded at both frequencies, 2.47 Hz and 4.98 Hz. This session on day one took approximately three hours total. 24-48 h later, participants were asked to return to the lab and complete the retention (4-blocks) test of the force-matching task (FMT), which took approximately 10-min total. The second day was limited to behavioral measures, meaning SEPs and EEG were not recorded on the second day, as previous research has shown that the majority of early corticospinal changes occur during the first day of early motor learning (Holland et al., 2015).

### 2.4. Novel force-matching tracking task

Participants were instructed to complete a novel motor task that required them to modulate the force from their right thumb in order to accurately match a waveform trace that varied in force, based upon a percentage of their individual maximal voluntary contraction (MVC) of the Abductor Pollicis Brevis (APB) muscle. Individual MVCs were determined by asking participants to apply as much force as possible onto the 50 kg force transducer, using their right thumb, while limiting any forearm, elbow, or shoulder integration (i.e., isolating the contraction to APB). MVCs were calculated based on the average of three trials. During the force-matching task (FMT), each trace was presented on a computer monitor positioned in front of the participant. The force transducer was stabilized on a height-adjustable table to the right of the seated participant and table height was adjusted to a comfortable height for each participant.

**FIGURE 2:**
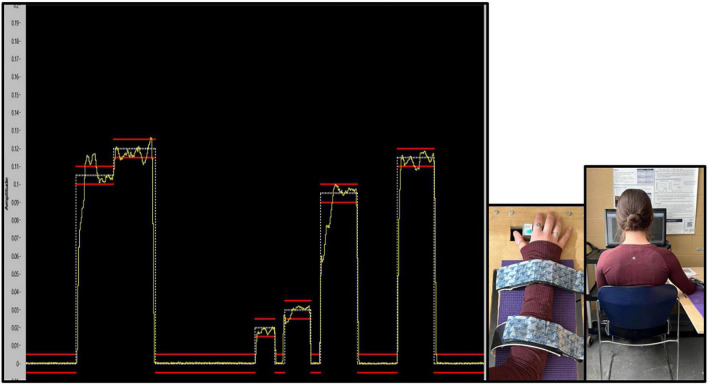
Absolute force variability (SD) for each phase of the force-matching task (FMT). Pre and post data were collected on the first day, and the retention test was completed 24–48 h later. Variability is presented as SD for force accuracy. Values represent mean ± SD.

The task was created and presented with a custom LABVIEW software program (National Instruments, Austin, TX, USA). To match the traces in the program, participants had to use their right thumb to push against a force transducer with a 50 kg load cell. The trace that participants were to match as accurately as possible was a continuous trace of white dots with two red error bars acting as a guide. The error bars were placed 0.05% above and below the dotted force trace. Throughout the duration of the task, participants were presented with augmented visual feedback in the form of a yellow solid line, representative of the force they were exerting against the transducer. This provided a visual depiction of how accurately they matched the intended trace. The traces varied between 2 and 12% of each participants Abductor Pollicis Brevis (APB) MVC (Ambalavanar et al., 2022), with isometric holds varying from 1 to 2.75 s in duration. This force-matching paradigm was developed based on previous literature (Pearce and Kidgell, 2010; Mehrkanoon et al., 2016; Dal Maso et al., 2018), and was designed to ensure that participants did not experience muscular fatigue. Please refer to Figure 2, depicting an example of what the participant saw while completing the force-matching task (FMT).

When completing the task, participants were seated in a standard stationary work chair with feet flat on the floor. Their right arm and hand were pronated and resting on the table, with their right thumb to the left of the force transducer. Their forearm was strapped to the table using two Velcro straps. This was to limit the involvement of the shoulder and elbow in the task, assisting in ensuring participants were limited to using their right thumb. The task was completed in the following order: pre/baseline (4-blocks of traces), acquisition (12-blocks of traces), and post (4-blocks of traces), 24-48 h later participants returned to the lab and completed the retention (4-blocks of traces) test. Each block consisted of three to five trials, and each trial was 20 s long. Blocks, which were a series of three to five trials, were presented in a randomized order for each participant, to ensure that there was not an order effect on performance and learning.

### 2.5. SEPs stimulating and recording parameters

As it pertains to peripheral SEPs, recording electrodes were placed according to the International Federation of Clinical Neurophysiologists (IFCN) guidelines (Nuwer et al., 1994). Surface EMG electrodes (Ag-AgCl, Meditate, conductive adhesive hydrogel) were placed on the ipsilateral brachial plexus (Erb’s point), allowing for the recording of the N9 SEP peak (Rossi et al., 2003). The Erb’s point electrode was referenced to the ipsilateral earlobe using electrode paste and an ear clip (Rossi et al., 2003). An additional electrode was placed over the C5 spinous process for recording the N11 and N13 SEP peaks, and the anterior tracheal cartilage acted as a reference for the C5 electrode. Finally, a ground surface electrode was placed over the contralateral lateral 1/3 of the clavicle. Prior to electrode placement, each site was cleaned and prepared by shaving, abrading using abrasive tape, and cleaned with an alcohol swab. Impedance was checked for peripheral electrodes; all signals had an impedance below 5.0 KΩ.

The following SEP peaks were identified and the amplitude was recorded and analyzed at baseline and post motor acquisition. Each participant’s “post” measurement was normalized to their baseline value, allowing for an assessment of proportional change in SEP peak amplitudes following motor acquisition. The peripheral N9 and the spinal N11 and N13 were each recorded using Signal4 Software (Version 4.08, Cambridge Electronic Design, Cambridge, UK), and the following peaks were recorded using a Waveguard™ whole-head high-density 64-electrode EEG cap (ANT Neuro, Netherlands), including the far-field N18 (P14–N18 complex), the parietal N20 (P14–N20 complex), and P25 (N20–P25 complex), the frontal N24 (P22–N24 complex), and the frontal N30 (P22–N30 complex). Each of these SEP peaks are reflective of activity within specific neural generators (Passmore et al., 2014).

SEPs were stimulated at two different sampling frequencies, this was to allow for the clear identification of the N24 SEP peak. The 2.47 Hz frequency was used to clearly identify the N30 peak, whereas the faster stimulating frequency at 4.98 Hz results in the attenuation of the N30 SEP peak, allowing for a clear identification of the N24 peak (Haavik and Murphy, 2013). The slow stimulation, 2.47 Hz, takes approximately 10 min, while the faster stimulation frequency at 4.98 Hz, takes approximately 5 min. Therefore, each round of SEP stimulations and EEG recording took approximately 15 min. Each stimulation frequency took place for 1,000 sweeps, allowing for a clear average of each SEP peak. Each stimulation frequency occurred twice, once prior to the novel motor tracing task and once after performing the force-matching task (FMT). While SEP stimulation occurred, participants were instructed to sit still in a standard office chair, with their feet flat on the floor, in a comfortable posture that they could maintain throughout the collection. The room remained quiet during this time.

#### 2.5.1. Stimulation parameters

Median nerve SEPs were elicited via stimulation of the median nerve over the right wrist, just proximal to the distal crease of the wrist. Stimulation intensity was set at motor-threshold of the Abductor Pollicis Brevis (APB) muscle for each participant, which was observed as the lowest possible intensity where a 1 cm visible thumb twitch occurred. This motor response occurred as a result of the electrical stimulation of the median nerve, as the median nerve is a mixed-nerve. This ensured that the 1a afferents were being stimulated, which will result in the short-latency SEP peaks, due to their projection to the cerebral cortex (Gandevia et al., 1984). For the stimulating electrodes, the anode was placed proximal in relation to the wrist while the cathode was placed distal in relation to the wrist (Andrew et al., 2018; MacDonald et al., 2019). SEP stimuli were sent via a Digitimer, and were electrical square pulses that were 200 μs in duration, delivered at a constant intensity, at frequencies of both 2.47 Hz and 4.98 Hz through Ag/AgCl EMG conductive adhesive surface electrodes (Meditrace™ 130, Kendall, and Mansfield, MA, USA). 1,000 sweeps for each stimulation frequency were delivered and were subsequently averaged.

### 2.6. EEG recording parameters

A Waveguard™ 64-electrode whole-head EEG cap (ANT Neuro, Netherlands) was used to record central SEP peaks, including the N18, N20, P25, N24, and N30. The Waveguard™ cap was connected to a TMSi REFA-8 amplifier with 64 EEG channels, four bipolar channels, and four auxiliary channels. The collection was run through asaLab™ (Netherlands), and collections were recorded at a sampling frequency of 2048 Hz (Navid et al., 2019). SEP analysis was completed on a separate laptop using Advanced Source Analysis (ASA™; Netherlands) and SPSS^®^ (Armonk, New York, NY, USA).

### 2.7. Data processing

#### 2.7.1. Force data

A custom LabVIEW™ program was used to filter and analyze the force data. A 0.5 s moving average window was applied to the data for smoothing of the force signal (Sonne and Potvin, 2015). Variables that were assessed include average absolute percent error and standard deviation of error as a measure of force variability. Error was assessed by comparing the participant’s force output to the force trace template target. Performance measures reported include both absolute values and those that have been normalized to each participant’s baseline score on the force-matching task (FMT).

To calculate percent error, the following equation was used:


$$Absolute\%Error=\left(\left(\left(\frac{ParticipantForceTrace}{ForceTrace}\right)x100\right)-100\right)$$

(Ambalavanar et al., 2022).

#### 2.7.2. SEPs

The SEPs signals were amplified (gain of 10,000) and filtered (0.2–1,000 Hz) on a laboratory computer (Andrew et al., 2018; Zabihhosseinian et al., 2021). Peripheral SEPs were recorded and analyzed in Signal4 software (Version 4.08, Cambridge Electronic Design, Cambridge, UK). This includes the peripheral N9, N11, and N13 peaks.

All SEP peaks were measured from the preceding trough/peak to the following peak/trough of interest. The change in amplitude in units of μV was recorded at baseline and at post measures. SEPs peak amplitude changes were then normalized to that peak’s baseline value for each participant. This allows for an assessment of proportional change for each SEP peak. Latency in units of ms for each peak was also recorded to ensure peaks were consistently identified for each participant.

To confidently say that SEP changes are not a result of peripheral changes, it is necessary to determine that the afferent input between pre and post measures was stable, to ensure any changes in central SEP peaks were a result of neural adaptations from learning and not a by-product of postural alterations, for example. This was done by ensuring stability of the N9 SEP peak over the brachial plexus/Erb’s point. The N9 had to remain stable pre-post to use the data set. Therefore, the N9 SEP peak had to be within ± 20% pre-post to include that participant’s neurophysiological data (Nuwer et al., 1994). All N9 SEP peaks met this inclusion criteria, and therefore no data sets were removed from analysis. SEP peaks were normalized to a participant’s baseline, i.e., a percentage of their pre-SEP peak amplitude, to account for differences in inter-participant baseline variability, allowing for comparisons between groups.

#### 2.7.3. EEG analysis

Whole-head EEG was used to record and analyze central SEP peaks, including the N18, N20, P25, N24, and N30. Data was cleaned and any artifacts, including eyeblinks, were removed prior to running analyses. Artifacts which were a result of muscle activity and ocular activity were removed using ASA software, excluding signals that were ± 100 μV. EEG data was filtered using a band-pass filter with a low cut-off of 0.2 Hz and a high cut-off of 1,000 Hz, slope steepness was set at 24 dB/octave (Andrew et al., 2018; Zabihhosseinian et al., 2021). Data was then averaged, providing averaged 64-electrode signals to obtain central SEP peak amplitudes and latencies. For each SEP peak, greater amplitudes are seen over electrodes closest to the neural generator responsible for that peak (Valeriani et al., 1998; Zabihhosseinian et al., 2021). Therefore, the N18 was recorded over the ipsilateral FC2 electrode, the N20 and P25 over the contralateral CP3, and the N24, and N30 over the contralateral FC1 electrode.

### 2.8. Statistical analyses

Statistical significance was set at *p* ≤ 0.05 for all analyses (SPSS v.24, IBM Corporation, Armonk, NY, USA). Effect sizes are reported using partial eta squared (η2), with a small effect as 0.01, medium as 0.06, and a large effect as 0.14 (Richardson, 2011). All numeric values are expressed as mean ± standard deviation (SD), unless otherwise stated. Normality was tested using Shapiro–Wilk’s test and Levene’s test was used as an assessment of homogeneity of variance.

#### 2.8.1. Behavioral

Motor performance was compared between and within groups. This was done for pre-acquisition, post-acquisition, and retention. A 2 × 3 mixed-design ANOVA with repeated measures of time (pre, post, and retention) and between subject factor of group (ADHD and control) as measures was performed on both the mean percent error and the force variability (SD). This was performed on both the absolute and normalized data, as the absolute data can show differences in absolute motor performance, whereas the normalized data can show performance improvements relative to baseline as a result of learning. Behavioral data was normally distributed, with the exception of the absolute “pre” scores for both groups. Therefore, log transformations were performed on the absolute performance scores to correct for this violation of normality.

#### 2.8.2. Neurophysiological

Neural adaptations were compared between groups using a 2 × 2 mixed-design ANOVA with the factor of time (pre-acquisition vs. post-acquisition) as the repeated measure and group (ADHD and control) defined as the between subject factor for each SEP peak. All SEP peak data was normally distributed. Furthermore, the Benjamini-Hochberg test was performed to correct for multiple comparisons that are independent from one another for short-latency cortical SEP peaks (Hochberg, 1988; Benjamini and Hochberg, 1995). Differences were hypothesized to be present in SEP peaks related to SMI, and therefore the Benjamini-Hochberg test was performed on the cortical SEP peaks including the N18, N20, P25, N24, and N30. This was done as each SEP peak is reflective of activity within unique neural generators (Passmore et al., 2014). This controls for a false discovery rate, and takes place by ranking each *p*-value from the smallest value to the largest value, which is done for all SEP peaks. These values are then compared to the Benjamini-Hochberg critical value (Hochberg, 1988; Benjamini and Hochberg, 1995). A given data set is then considered statistically significant if the adjusted *p*-value is smaller than the family-wise error rate or false discovery rate of 0.20. The family-wise error rate of 0.20 was established to reduce the chance of a type-II error, and thus reduce the chance of missing valuable information. The *p*-values reported are the original unadjusted *p*-values, as per recommendations within the literature (McDonald, 2009). However, each value was checked using the Benjamini-Hochberg correction as described, and were only reported if they continued to meet statistical significance. The spinal N11 and N13 were collected to confirm that there were not any differences at the spinal level, and hence are reported separately.

## 3. Results

### 3.1. Behavioral

Supplementary data can be found in Supplementary Table 1.

#### 3.1.1. Mean percent error

**FIGURE 3:**
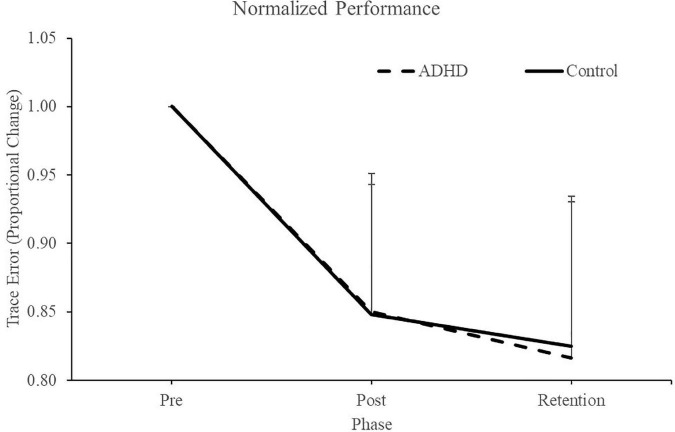
Normalized performance scores for each phase when assessing trace error. Pre and retention measures have been normalized to each individuals baseline (pre) score. Retention data was collected 24-48 h after the pre and post data. ADHD scores are in the dashed line, controls are in the solid line. Values represent mean ± SD.

**FIGURE 4:**
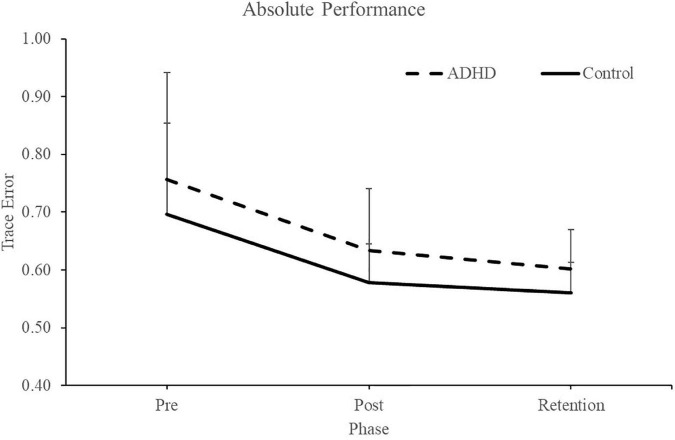
Depiction of the custom force-matching task (FMT) program on LABVIEW as participants view it. The yellow line indicates the participants force output on the transducer. The white line is the intended trace, while red lines reflect boundary guides. The *y*-axis of the program is the amplitude of the trace, which is calibrated as a percent (%) of each participants MVC, and vary from 2 to 12% MVC. The *x*-axis is the trace as it progresses through time from 0 to 20s.

Normalized performance scores can be seen in Figure 3 and Table 1. There was a significant effect of time (F_2,28_ = 61.645; *p* < 0.0001; partial η^2^ = 0.688) for the normalized performance scores. This shows that both groups (ADHD and control) improved from baseline to post-measures (ADHD: 0.850 ± 0.093 vs. control: 0.848 ± 0.103) and at retention compared to baseline (ADHD: 0.816 ± 0.114 vs. control: 0.825 ± 0.110). Post-hoc tests showed that pre-scores were significantly different than retention and post, while retention and post were not significantly different from one another. An effect of group was not present (F_1,28_ = 0.008; *p* = 0.929; partial η^2^ = 0.000). *Absolute mean scores:* Absolute performance scores can be seen in Figure 4 and Table 1. There was a significant main effect of time (F_2,26_ = 33.759; *p* < 0.0001; partial η^2^ = 0.650) for absolute performance scores. This illustrates that both groups (ADHD vs. control) improved from baseline (ADHD: 0.757 ± 0.184 vs. control: 0.696 ± 0.158) to post-measures (ADHD: 0.633 ± 0.107 vs. control: 0.578 ± 0.068) and from baseline to retention (ADHD: 0.601 ± 0.068 vs. control: 0.560 ± 0.052). Post-hoc tests showed that pre-scores were significantly different than retention and post, while retention and post were not significantly different from one another. An effect of group (ADHD vs. control) was not reached (F_1,26_ = 2.036; *p* = 0.137; partial η^2^ = 0.077).

**TABLE 1:** Illustrating normalized/absolute performance values for the novel force-matching task (FMT).

Percent error (Normalized/Absolute)	Pre	Post	Retention
ADHD	1	0.85 ± 0.09	0.82 ± 0.11
	0.76 ± 0.18	0.63 ± 0.11	0.60 ± 0.07
Control	1	0.85 ± 0.10	0.83 ± 0.11
	0.70 ± 0.16	0.58 ± 0.07	0.56 ± 0.05

Values represent mean ± SD.

#### 3.1.2. Force variability (SD)

**FIGURE 5:**
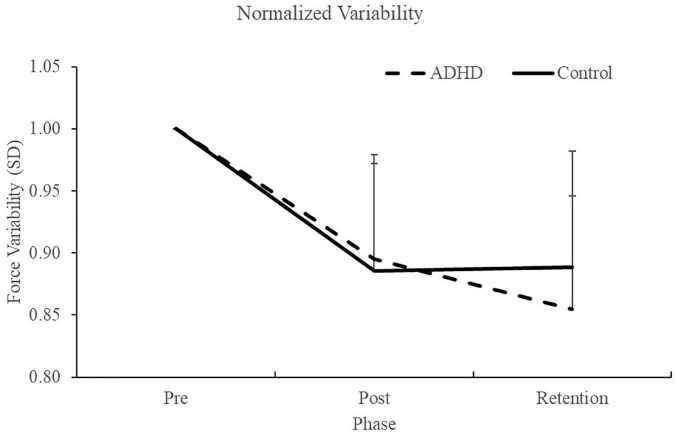
Normalized force variability, when assessing SD, for each phase of the force-matching task (FMT). Variability is presented as SD of the force trace accuracy. Retention tests occurred 24–48 h after the pre and post data were collected. Values represent mean ± SD.

**FIGURE 6:**
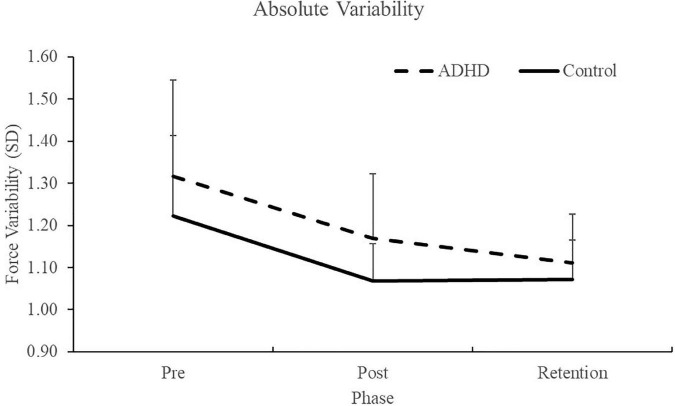
Absolute (raw) performance scores for each phase when assessing trace error. ADHD scores are in the dashed line and controls are in the solid line. Pre and post measures were collected on day one and the retention test occurred 24–48 h later. Values represent mean ± SD.

Normalized force variability data can be seen in Figure 5 and Table 1. There was a significant effect of time (F_2,28_ = 46.446; *p* < 0.0001; partial η^2^ = 0.624) for the normalized force variability. This shows that both groups (ADHD and control) became less variable from baseline to post-measures (ADHD: 0.90 ± 0.077 vs. control: 0.89 ± 0.093) and at retention compared to baseline (ADHD: 0.85 ± 0.091 vs. control: 0.89 ± 0.093). Post-hoc tests showed that variability at pre-measures were significantly different than retention and post, while retention and post were not significantly different from one another. An effect of group was not present (F_1,28_ = 0.153; *p* = 0.698; partial η^2^ = 0.005). *Absolute variability:* Absolute variability can be seen in Figure 6 and Table 1. There was a significant main effect of time (F_2,26_ = 42.168; *p* < 0.0001; partial η^2^ = 0.601) for absolute variability. This illustrates that both groups (ADHD vs. control) had less variable force output from baseline (ADHD: 0.1.32 ± 0.23 vs. control: 1.22 ± 0.19) to post-measures (ADHD: 1.17 ± 0.15 vs. control: 1.07 ± 0.089) and from baseline to retention (ADHD: 1.11 ± 0.12 vs. control: 1.07 ± 0.093). *Post-hoc* tests showed that variability at pre-measures were significantly different than retention and post, while retention and post were not significantly different from one another. An effect of group (ADHD vs. control) was not reached (F_1,26_ = 2.820; *p* = 0.104; partial η^2^ = 0.091), although a medium effect size was present.

### 3.2. Neurophysiological SEPs data

**FIGURE 7:**
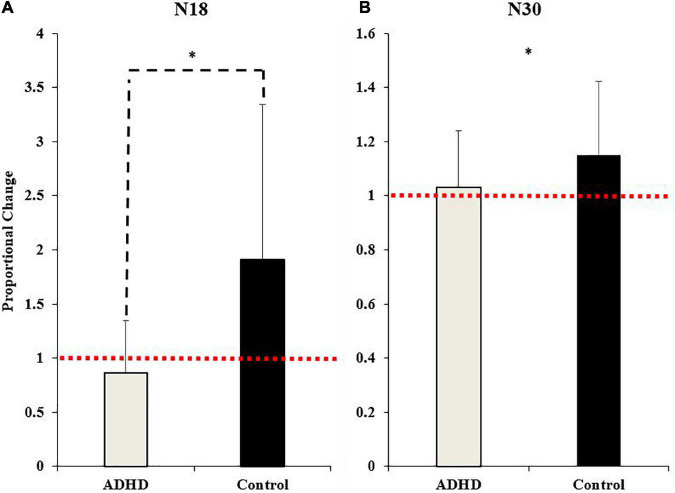
Normalized SEP peak amplitudes relative to baseline (red dotted line) for the N18 **(A)** and the N30 **(B)**. ADHD participants are in gray and controls are in black. Values represent mean ± SD. Dashed bars (- - -) and asterisks (*) denote significant group interactions, and asterisks (*) only indicate significant effects of time.

All participant’s SEP data was included, as the inclusion criteria of the N9 SEP peak was always met. Specifically, the N9 was recorded over the ipsilateral brachial plexus, and when comparing pre-post measures, the N9 differed by no more than ± 20% from baseline measures (Nuwer et al., 1994; Zabihhosseinian et al., 2021). This assessment is done to ensure that any central SEP peak changes are not inadvertently a result of peripheral changes, such as to posture. This was also confirmed statistically, where the N9 had no effect of time (F_1,28_ = 0.015; *p* = 0.903; partial η^2^ = 0.001) or group present (F_1,28_ = 0.059; *p* = 0.811; partial η^2^ = 0.002). Therefore, all participants data is included in the SEP peak analysis. SEP peak data can be seen below in Figure 7.

#### 3.2.1. Spinal SEP peaks

##### 3.2.1.1. N11

No effect of time (F_1,28_ = 3.523; *p* = 0.071; partial η^2^ = 0.112) or group were present (F_1,28_ = 0.000; *p* = 0.984; partial η^2^ = 0.000), although a medium effect size was reached for time.

##### 3.2.1.2. N13

No effect of time (F_1,28_ = 0.990; *p* = 0.328; partial η^2^ = 0.034) or group were present (F_1,28_ = 0.068; *p* = 0.797; partial η^2^ = 0.002).

#### 3.2.2. Cortical SEP peaks

##### 3.2.2.1. N18

A main effect of time was not present (F_1,28_ = 4.035; *p* = 0.054; partial η^2^ = 0.126), although it approached significance and a medium effect size was evident. A significant effect of group (F_1,28_ = 7.212; *p* = 0.012; partial η^2^ = 0.205) and a time x group interaction were present (F_1,28_ = 7.212; *p* = 0.012; partial η^2^ = 0.205). The N18 decreased in those with ADHD (0.87 ± 0.48) and increased in controls (1.91 ± 1.43).

##### 3.2.2.2. N20

No effect of time (F_1,28_ = 0.048; *p* = 0.829; partial η^2^ = 0.002) or group were present (F_1,28_ = 0.888; *p* = 0.354; partial η^2^ = 0.031).

##### 3.2.2.3. P25

No effect of time (F_1,28_ = 0.379; *p* = 0.543; partial η^2^ = 0.013) or group were present (F_1,28_ = 2.367; *p* = 0.135; partial η^2^ = 0.078), although a medium effect size was present when comparing between groups.

##### 3.2.2.4. N24

No effect of time (F_1,28_ = 0.459; *p* = 0.504; partial η^2^ = 0.016) or group were present (F_1,28_ = 0.785; *p* = 0.383; partial η^2^ = 0.027).

##### 3.2.2.5. N30

**FIGURE 8:**
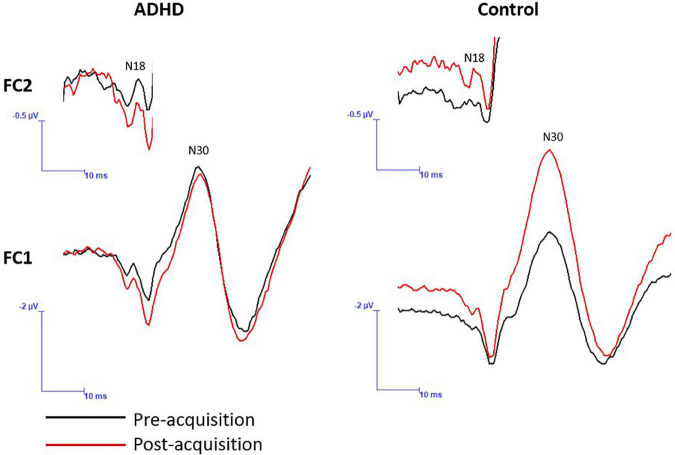
A representative dataset of cortical SEP peaks where significant changes were present. Both SEP peaks were collected at 2.47 Hz, and the N18 was collected at FC2 and the N30 at FC1. Pre-acquisition activity is in black and post-acquisition is in red.

A significant effect of time (F_1,28_ = 4.395; *p* = 0.045; partial η^2^ = 0.136) was present. The N30 increased in both groups (ADHD: 1.03 ± 0.21; controls: 1.15 ± 0.27). A main effect of group or a time by group interaction were not present (F_1,28_ = 1.815; *p* = 0.189; partial η^2^ = 0.061). Representative traces of SEP peaks where effects were present can be seen in Figure 8.

## 4. Discussion

This is the first work to assess a motor acquisition paradigm dependant on force modulation, in conjunction with neural markers in the form of SEPs, in young adults with ADHD. The pattern of results yielded indicate that there are differences in the way that those with ADHD process afferent information when learning a novel force-matching task (FMT) in a manner that differs from those in neurotypical controls. The specific SEP peaks that changed as a result of the novel force-matching task (FMT) were the N18 and the N30. The N18 SEP peak, which reflects activity within cortico-cerebellar networks, increased in the control group, yet decreased in ADHD group. In contrast, the N30 SEP peak increased in both those with and without ADHD, suggesting similar changes in each group. Additionally, both groups exhibited improvements in performance at post and retention measures, suggesting that they did indeed learn throughout the acquisition phase of the paradigm. When assessing absolute performance measures, those in the ADHD group appeared to exhibit greater error at all phases of the paradigm, when compared to controls, although this did not reach statistical significance a medium effect size was present, and may be an important topic of inquiry in the future. Therefore, the normalized performance scores suggest that both those with and without ADHD learned to a similar extent, as seen by similar proportional improvement (i.e., reduction in error), although absolute values may suggest reduced overall performance in those with ADHD when compared to neurotypical controls. Overall, the opposite direction of change in the N18 SEP peak neural marker following acquisition of the force-matching task, coupled with the lack of group differences in the performance of the task, suggests that adults with ADHD did learn the force task, but did so using a different pattern of neural processing.

### 4.1. Behavioral data

One way to infer if motor learning has occurred can be via changes in performance over time, such as improvement in accuracy or reduced variability after the acquisition of a novel skill (Schmidt and Lee, 2005). Throughout the acquisition of a new motor skill there will be a progressive refinement in motor performance. Additionally, the consolidation of a skill, observed via maintained or further improvement in performance at retention measures, can be assessed. In addition to accuracy, the level of force-variability can provide insight into the degree of learning as more skilled performers can produce the forces more consistently (i.e., with lower variability) (Selen et al., 2006). The results from the current work suggest that both those with and without ADHD learned the novel force-matching task (FMT). This was observed as both groups had improvements in on task performance post-acquisition and at retention when compared to their baseline performance scores. In addition, both groups exhibited reduced variability at post-acquisition and retention. Together these results suggest that both groups learned the task to a similar extent, as both groups exhibited approximately 15% less error post-acquisition when their post scores were normalized to their baseline. Furthermore, when completing their retention test, scores remained similar to their post-acquisition measures, if not ever so slightly improved, as error was approximately ∼18-19% less than that of their baseline measures. Previous research has noted similar improvements in performance in response to learning a motor task dependent on force modulation (Ambalavanar et al., 2022). In the current study, similar results were present when assessing absolute performance scores, although those with ADHD had increased absolute error and variability at each phase when compared to neurotypical controls. This may suggest that those with ADHD experience more difficulty with motor tasks dependent on force modulation and proprioception, when compared to neurotypical controls. Potential differences with proprioceptive weighting, such as those suggested here, may be related to the reduction in the N18 in those with ADHD noted in the current study. The reduction in the N18, which is likely reflective of reduced inhibition of dorsal column nuclei, inferior olives, and cortico-cerebellar networks (Noël et al., 1996; Sonoo, 2000; Rossi et al., 2003; Haavik and Murphy, 2013; Andrew et al., 2018), may be a result of those with ADHD experiencing difficulty with the relative weighting of the visual versus force and proprioceptive feedback of the task. This change in relative weighting results in a greater reliance on the proprioceptive sensory afferents during the learning process, thus reducing the filtering effect to allow for the fine tuning of force-modulation to accurately meet the demands of the task.

### 4.2. Neurophysiological SEPs data

**FIGURE 9:**
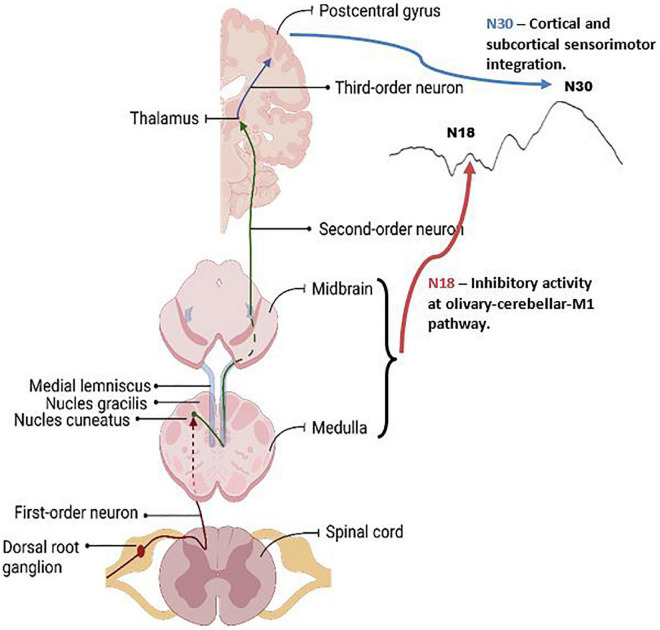
Graphic of the neuroanatomy and neural generators associated with the SEP peaks that exhibited changes in this paper, the N18 and N30 peaks. The N18 is suggested to reflect inhibitory activity within olivary-cerebellar-M1 locations, and the N30 reflects cortical and subcortical activity associated with sensorimotor integration. Graphic was created with BioRender.com.

SEPs offer a non-invasive technique to assess cortical and sub-cortical processing between groups and in response to various tasks. Each SEP peak is reflective of activity within a specific neural structure (Passmore et al., 2014). Due to this, they provide an invaluable technique allowing for the assessment of the neural processes related to motor learning and SMI. Previous research has shown distinct SEP peak changes in response to visuomotor tasks and in many populations, including those experiencing fatigue and individuals with subclinical neck pain (SCNP), further validating their relevance associated with the interpretation of neural correlates of motor learning (Andrew et al., 2018; Zabihhosseinian et al., 2021). The current study is the first, to our knowledge, to use SEPs to aid the assessment of force-dependent motor learning in those with ADHD, thus providing novel insight into the neural underpinnings of motor learning in this population. The current study yielded results suggesting that two SEP peaks in particular had significant changes between groups or after motor acquisition, these peaks are the N18 and N30. Figure 9 depicts the underlying neural activity associated with these pathways. The lack of within or between group differences in the other SEP peaks assessed, including the N20 and N24, may be a result of the specific motor paradigm used, which had differential weighting of visual and proprioceptive afferent input when compared to a visuomotor tracing task, which previous literature has shown to result in changes to these SEP peaks in control populations (Andrew et al., 2018; O’brien et al., 2020). Due to the limited literature utilizing SEPs as a measure of neural processing in those with ADHD, it is difficult to contrast the lack of differences in these peaks from the current study to prior research. Therefore, the discussion will focus on the SEP peaks which demonstrated changes in the current work, namely the N18 and N30.

#### 4.2.1. N18

The results from the current study showed that those with ADHD exhibited different neural processing after learning the novel force-matching task (FMT) than did controls. This difference was evident for the N18 SEP peak, where those in the ADHD group exhibited a reduction in the N18 peak amplitude after performing the motor acquisition paradigm and controls exhibited an increase in peak amplitude. This is the first study to utilize a motor acquisition task highly dependent on proprioception and force modulation in those with ADHD, in conjunction with a neural measure to assess cortical and subcortical processing. The current work is in line with previous research utilizing similar methodology in controls, which saw as an increase in the N18 SEP peak in controls after acquisition of a novel force-matching task (FMT) (Ambalavanar et al., 2022). The N18 is recognized as having neural generators within the brainstem, in particular between the lower medulla and the midbrain pontine regions (Sonoo et al., 1991; Noël et al., 1996; Haavik and Murphy, 2013). Furthermore, the N18 is reflective of inhibitory activity at the level of the medulla, as a result of activity within the dorsal column medial lemniscus nuclei (Noël et al., 1996; Sonoo, 2000; Rossi et al., 2003). However, the N18 is also a marker of activity generated within the cuneocerebellar tract, the cerebellum, and inferior olive, this being as a result of collaterals diverging from the medial lemniscus within the medulla (Noël et al., 1996). The cuneate nuclei relay both cutaneous and proprioceptive information to the thalamus, and then towards the cerebral cortex, in addition to its role in feed-back regulated cerebellar SMI (Hand and Van Winkle, 1977; Marshall, 1984; Berkley et al., 1986; Pascual-Leone and Torres, 1993; Haavik and Murphy, 2013). This posits that the N18 reflects alterations to cerebellar activity, such as activity related to cerebellar SMI (Haavik and Murphy, 2013). The increased N18 in controls at post measures has been noted in prior literature utilizing a similar motor paradigm (Ambalavanar et al., 2022). This may reflect increased inhibitory activity in olivary-cerebellar-M1 networks, suggestive of greater dependence on cerebellar SMI and inhibitory activity for feedback during processing related to force modulation in the thumb, to control force output during this task as a result of learning (Ambalavanar et al., 2022). Therefore, the differences in the N18 in those with ADHD compared to controls may suggest differences in the olivary-cerebellar-M1 processing in response to learning the novel force-matching task (FMT).

The cerebellum is a neural structure that plays a fundamental role in the process of learning. Particularly, the cerebellum has increased activity during the initial stages of learning (Jenkins et al., 1994; Eliassen et al., 2001; Doyon et al., 2002; Penhune and Doyon, 2002; Floyer-Lea and Matthews, 2005; Gao et al., 2012; Baarbé et al., 2014). Consequently, reduced inhibition of the cerebellum to the primary motor cortex is noted after exposure to a novel motor task (Baarbé et al., 2014). Therefore, the novel finding from the current study, that those with ADHD had a reduction in the N18, may be reflective of reduced inhibition or inhibitory activity of olivary-cerebellar-M1 activity after performance of the force-matching task (FMT). This may suggest that those with ADHD had difficulty processing the proprioceptive input when learning this task, seen as a reduction in the filtering effect in order to continually refine motor output via proprioceptive and force feedback (Haavik and Murphy, 2013). Previous work illustrates that there are proprioceptive deficits in those with ADHD (Goulardins et al., 2013; Jung et al., 2014; Alba et al., 2016; Sanz-Cervera et al., 2017). Previous literature has noted that when a novel task is not learned well, this will result in increased activity within cerebellar brain regions (Doyon et al., 2002; Floyer-Lea and Matthews, 2005; Manzoni, 2007; Dancey et al., 2016; Andrew et al., 2018). This is possibly a result of ongoing error correction during the acquisition phase. Therefore, the proprioceptive and motor control deficits in those with ADHD are likely related to the N18 reduction that was found in the current study. It follows that the N18 reduction may have occurred due to an enhanced reliance on, or difficulty processing proprioceptive feedback if this proprioceptive processing is indeed altered in adults with ADHD.

Previous work, utilizing a 20 min repetitive typing task, found an decrease in the N18 in controls, which was suggestive of a reduction in a filtering effect prior to cortical processing during the early stages of learning (Haavik and Murphy, 2013). The results from the current study suggest that those in the ADHD group may experience difficulty with the proprioceptive-centric demands of the current task, resulting in an enhanced dependence on force-modulation afferents via error monitoring, reflecting reduced inhibitory activity at the level of the cerebellum as a reduction in the N18 post motor acquisition (Andrew et al., 2018). Interestingly, McCracken et al. (2022) found contrasting results in those with ADHD when performing a motor acquisition paradigm utilizing different task demands. When completing a visuomotor task, those with ADHD had an increase in the N18, whereas controls had a decrease (McCracken et al., 2022). It is likely that the different task demands in the current study, which were heavily dependent on force-modulation and proprioception, resulted in the reduced N18 in those with ADHD. Therefore, this finding suggests that tasks that are heavily dependent on force-modulation via proprioception result in reduced inhibitory activity in those with ADHD, when compared to neurotypical controls.

#### 4.2.2. N30

The current study noted an increase in the N30 in both groups after completing the novel force-matching task (FMT). Previous work has found that motor acquisition paradigms result in an increase in the N30 SEP peak amplitude (Andrew et al., 2015a,b; Zabihhosseinian et al., 2020). Recent work utilizing a novel force-matching task (FMT) found an increase in the N30 SEP peak after the learning paradigm was complete (Ambalavanar et al., 2022). The N30 SEP peak is reflective of activity at both cortical and sub-cortical levels, including the basal ganglia, thalamus, pre-motor areas, primary motor cortex, and the supplementary motor area (Rossini et al., 1987; Kaňovský et al., 2003; Rossi et al., 2003; Cebolla and Chéron, 2015). This peak is generally thought to reflect sensorimotor integration (Rossi et al., 2003). Source localization techniques have identified that the N30 neural generators have four distinct locations, including the contralateral primary somatosensory cortex, prefrontal cortex, cingulate, and bilateral secondary somatosensory cortex (Lelic et al., 2016). However, the prefrontal cortex is the neural source with the greatest activity during the N30 latency timeframe, and this region is related to SMI (Lelic et al., 2016). The increase in the N30 in the current study may reflect an upregulation of SMI neural processes, including those related to prefrontal function in response to task acquisition for both groups. Interestingly, the prefrontal cortex is one of the most commonly noted sites of neural alterations present in those with ADHD (Barkley, 1997; Sowell et al., 2003; Seidman et al., 2006).

The increase in the N30 in both groups suggests increased activity in brain regions heavily involved in SMI. Therefore, the demands of the novel force-matching task (FMT) resulted in similar activation patterns in these brain regions in those both with and without ADHD. Although of relevance, a main effect of group was absent when assessing the N30, a medium effect size does suggest that there may be differences in the N30 between groups. For instance, the control group saw a mean increase in the N30 by 15% at post measures, whereas those in the ADHD group exhibited a modest mean increase of 3%. This suggests that there may be inherent differences in SMI processes in those with ADHD when completing a proprioceptive dominant motor acquisition task, such as the one utilized in the current study. Specifically, an attenuated increase in the N30 when compared to neurotypical controls. If this is the case, it may be a result of altered neural structure and function in those with ADHD, such as those in prefrontal cortical regions (Barkley, 1997; Sowell et al., 2003; Seidman et al., 2006), affecting processes related to SMI. McCracken et al. (2022) also showed a reduction in the N30 in those with ADHD compared to a control group whose N30 SEP peak increased post motor-acquisition. In the future, incorporating further assessment techniques that are sensitive to neural activity, such as fMRI or source localization, as an initial cost-effective starting point, would elucidate the role of localized neural regions or structures in such processes, which may prove to be invaluable, to further enhance the understanding of how ADHD influences motor learning and SMI.

### 4.3. Limitations

Although pre-screening did include assessing for the presence of any known neurological conditions, DCD was not directly a part of that screening. This could pose a potential limitation, as recent work has suggested motor difficulties typically associated with the ADHD phenotype may be related to a co-occurring deficit in motor abilities (Farran et al., 2020). The ADHD group was able to improve their performance accuracy at similar rates to the non-ADHD group, however future studies assessing motor performance in ADHD should consider directly screening for DCD, such as by utilizing a questionnaire (Meachon et al., 2022), as the inclusion of even a small number of participants with DCD would increase the data variability and possibly result in type II errors. It should be noted that the data for some SEP peaks has large standard deviations due to between-subject variability. However, there is no way to avoid this as the way an individual’s gyri are folded determines the shape and amplitude of vector picked up by EEG electrodes. Additionally, the nature of the design and formatting of the force-matching task (FMT) may not have been optimal or conducive to motor learning, due to the slight delay between blocks/trials within LabVIEW. Thus, the discontinuous nature of the delivery may have made it more difficult, and limited further performance improvements at retention. However, it should be noted that the delay was minimal. In the future, creating a delivery method that limits any lag between blocks and trials, that can be delivered automatically and continuously through the software, will allow for a more streamlined or continuous motor task. In the future, including a transfer task that requires a similar set of skills, yet under a somewhat different set of sensory conditions, would allow for an assessment of how well the motor skill was truly acquired and can be transferred to a related task. For instance, a transfer task that lacks the yellow force-feedback line staying present on the screen, and is replaced by a cursor that only shows the current, and not past force-output or accuracy, would allow for the assessment of how those with ADHD perform a motor task that was learned, lacking visual knowledge of results during the performance.

## 5. Conclusion

This present work is the first to assess the neural mechanisms involved in force-dependent motor learning, heavily reliant on proprioception, in young adults with ADHD. The current technique involved the assessment of short-latency SEPs and behavioral improvements via performance accuracy. Those in the ADHD group exhibited a significantly reduced N18 SEP peak when compared to neurotypical controls whose N18 increased post-motor acquisition, suggesting reduced olivary-cerebellar-M1 inhibitory activity in the ADHD group, in response to the novel motor task. This may reflect an increased reliance on proprioceptive feedback via a reduction in the filtering effect in order to perform the task, potentially as a result of difficulty in the processing and integration of the force and proprioceptive input in association with the visual feedback presented. Behaviorally this may be related to the trend for increased absolute error in the ADHD group. However, both groups showed equivalent improvement in absolute and relative performance, thus task learning did occur to a similar degree in both groups. There was also a similar increase in the N30 peak, contrasted by the opposite adaptation of the N18 peak. In the future, utilizing forms of neural assessment that provide an improved form of spatial acuity, such as those offered by source localization techniques, or fMRI if feasible, could prove beneficial in improving our comprehension of how those with ADHD learn novel motor tasks, particularly those that require a high level of force modulation acuity. Overall, this present work suggests that adults with ADHD exhibit different neural processing related to learning a force-dependent motor paradigm.

## Data availability statement

The raw data supporting the conclusions of this article will be made available by the authors, without undue reservation.

## Ethics statement

The studies involving human participants were reviewed and approved by Ontario Tech University Research Ethics Board. The patients/participants provided their written informed consent to participate in this study.

## Author contributions

HM, BM, UA, and PY conception and design of research. HM and UA performed the experiments. HM analyzed the data and drafted the manuscript. HM, BM, PY, and UA interpreted the results. HM, BM, PY, and CG edited and revised the manuscripts and approved the final version of the manuscript. All authors contributed to the article and approved the submitted version.
